# Clinical Applications of Nasal Nitric Oxide in Allergic Rhinitis: A Review of the Literature

**DOI:** 10.3390/jcm12155081

**Published:** 2023-08-02

**Authors:** Giuseppina Marcuccio, Pasquale Ambrosino, Claudia Merola, Fabio Manzo, Andrea Motta, Gaetano Rea, Elena Cantone, Mauro Maniscalco

**Affiliations:** 1 Istituti Clinici Scientifici Maugeri IRCCS, Pulmonary Rehabilitation Unit of Telese Terme Institute, 82037 Telese Terme, Italy; giuseppina.marcuccio@icsmaugeri.it (G.M.); claudia.merola@icsmaugeri.it (C.M.); 2 Istituti Clinici Scientifici Maugeri IRCCS, Directorate of Telese Terme Institute, 82037 Telese Terme, Italy; pasquale.ambrosino@icsmaugeri.it; 3 Fleming Clinical Laboratory, 81020 Casapulla, Italy; fabioce26@gmail.com; 4 Institute of Biomolecular Chemistry, National Research Council, 00185 Pozzuoli, Italy; andrea.motta@icb.cnr.it; 5 Department of Radiology, Monaldi Hospital, AO dei Colli, 80131 Naples, Italy; 6 Department of Neuroscience, Reproductive and Odontostomatological Sciences—ENT Section, University of Naples Federico II, 80138 Naples, Italy; elena.cantone@unina.it; 7 Department of Clinical Medicine and Surgery, University of Naples Federico II, 80138 Naples, Italy

**Keywords:** allergy, rhinitis, chronic respiratory disease, rehabilitation, exercise, disability, outcome

## Abstract

Allergic rhinitis, a common allergic disease affecting a significant number of individuals worldwide, is observed in 25% of children and 40% of adults, with its highest occurrence between the ages of 20 and 40. Its pathogenesis, like other allergic diseases, involves innate and adaptive immune responses, characterized by immunologic hypersensitivity to environmental substances. This response is mediated by type 2 immunity. Within type 2 allergic diseases, certain molecules have been identified as clinical biomarkers that contribute to diagnosis, prognosis, and therapy monitoring. Among these biomarkers, nitric oxide has shown to play a key role in various physiological and pathological processes, including neurotransmission, immunity, inflammation, regulation of mucus and cilia, inhibition of microorganisms, and tumor cell growth. Therefore, measurement of nasal nitric oxide has been proposed as an objective method for monitoring airway obstruction and inflammation in different settings (community, hospital, rehabilitation) and in various clinical conditions, including upper airways diseases of the nose and paranasal sinuses. The purpose of this review is to analyze the potential mechanisms contributing to the production of nasal nitric oxide in allergic rhinitis and other related health issues. Additionally, this review aims to identify potential implications for future research, treatment strategies, and long-term management of symptoms.

## 1. Introduction

Allergic rhinitis (AR) is the most common allergic disease worldwide, impacting approximately 400 million people [1,2,3]. Over the past 30 years, its prevalence has significantly increased, affecting 25% of children and 40% of adults [2,3,4]. This rise can be attributed to the effects of urbanization and heightened levels of pollutants, which exacerbate pollen sensitization [2,3,4]. Symptoms of AR typically manifest in childhood, adolescence, and early adulthood, peaking between the ages of 20 and 40 [3,4]. In preschool-age children, AR has a notable incidence of 17.9%, with a higher prevalence among males [4,5,6].

The pathogenesis of AR, like other allergic diseases, involves innate and adaptive immune responses, characterized by immunologic hypersensitivity to environmental substances [2,3,4]. This response is mediated by type 2 immunity, which involves T-helper 2 (Th2) cells, eosinophils, mast cells, and M2 macrophages [7,8]. According to the Allergic Rhinitis and its Impact on Asthma (ARIA) guidelines [2,9], the clinical diagnosis of AR is based on positive skin-prick testing for allergens or serum immunoglobulin E (IgE) tests. AR symptoms, including sneezing, nasal obstruction, itching, and rhinorrhea triggered by allergen exposure, can also be associated with other conditions such as asthma, rhinosinusitis, otitis media, and conjunctivitis, leading to clinical complexities in management and treatment [2]. These complications also contribute to a decreased quality of life (QoL) and substantial healthcare costs, amounting to billions of dollars in the United States [10,11].

In the era of precision medicine, certain molecules have been identified as key biomarkers in the pathogenesis of AR, providing crucial information for precise diagnosis and treatment monitoring [7]. These biomarkers may help identify disease subtypes (endotypes) and clusters, guiding targeted interventions and monitoring treatment effectiveness [7]. In this regard, nitric oxide (NO) has been proposed as the most relevant biomarker of type 2 allergic diseases, including AR [7]. NO is an inflammatory mediator and, therefore, it has been extensively studied in various clinical conditions. Thus, measurement of fractional exhaled NO (FeNO) has become a useful tool for monitoring inflammatory diseases of lower airways, such as bronchial asthma [12]. Similarly, nasal NO (nNO), which plays a significant role in physiological and pathological processes like neuro-transmission, immunity, inflammation, and mucociliary regulation [13], has been proposed as an objective measure for monitoring upper airway inflammation [14,15]. However, the relationship between AR and nNO remains controversial, with conflicting findings in the scientific literature [16,17].

The objective of this review is to explore the potential mechanisms involved in nNO production in AR and their associations, particularly in the presence of comorbidities. The review aims to identify implications for pathophysiological mechanisms, treatment strategies, and long-term symptom management in AR.

## 2. Nasal Nitric Oxide

### 2.1. Sources and Biological Mechanisms

NO is a small diatomic molecule weighing 30 Da, known for its high reactivity caused by its unpaired electron, thus requiring precise enzymatic control for both activation and inactivation [15].

This molecule is synthesized by several forms of NO synthase (NOS) from the semi-essential amino-acid L-arginine and oxygen (O_2_) [13,18]. Three isoforms of NOS have been identified, two of which are constitutive, namely neuronal (nNOS) and endothelial (eNOS), while the other one is inducible (iNOS) because is produced in activated cells [18]. Although being inducible, iNOS acts as a constitutive form in paranasal sinuses [18]. Under normal circumstances, modest quantities of NO are produced by the constitutive enzymes nNOS and eNOS, which rely on intracellular calcium signals in neurons or smooth cells [18]. These signals can arise from events such as an action potential occurring at a nerve ending or the stimulation of endothelial cell receptors by acetylcholine [19]. Instead, iNOS produces large amounts of NO, being induced by inflammatory cytokines, including tumor necrosis factor-α (TNF-α) and interleukin-1β (IL-1β), by means of activation of transcription factors, such as nuclear factor κB (NF-κB) [18]. The production of NO in paranasal sinuses, which can serve as a reservoir for NO, can also be enhanced by the activation of quorum-sensing pathways linked to microbial films [18]. 

The NO molecule derives from oxidation of extracellular L-arginine after its uptake. This pathway has been the target in several research for the development of NOS inhibitors, such as NG-monomethyl-L-arginine (L-NMMA) and NG-nitro-L-arginine-methyl-ester (L-NAME) [20,21]. These molecules interact with all NOS isoforms, while amino-guanidine and glucocorticoids have been proposed to be specific inhibitors of iNOS [20,21].

nNO plays different roles in many physiological and pathological processes. It inhibits the growth of microorganisms (bacteria, viruses, fungi) and tumoral cells [16]. Given its short half-life, which ranges from milliseconds to few seconds, NO is a potent vasodilator, and induces relaxation (vasodilation and bronchodilation) in smooth muscle cells through a mechanism that stimulates the production of cyclic guanosine monophosphate (cGMP) from guanosine triphosphate (GTP) via activation of the enzyme guanylate cyclase that decreases calcium levels [16,22]. At low concentration only, NO reacts with reactive radicals [15]. It is also involved in muco-ciliary function, increasing ciliary beating, and in immune response [15]. In this contest, several authors studied the effects of L-arginine on the muco-ciliary clearance, increasing ciliary beat frequency in vitro while sodium nitroprusside (SNP) stimulates muco-ciliary activity in the maxillary sinus [17,18,23].

Various functions of NO derive from its different concentrations [24,25]. A Th2-mediated response results in high levels of NO, which induce cellular apoptosis and downregulation of adhesion molecules [24,25]. Therefore, high and sustained levels of NO, resulting from the activation of iNOS, can exhibit both toxic and immune-regulating effects [25]. On the other hand, low levels of NO produced in a pulsatile manner through the activation of nNOS and eNOS can contribute to pro-inflammatory alterations [24,25], including vasodilatation and neutrophils migration.

Cells expressing iNOS include fibroblasts, natural killer cells (NK), chondrocytes and keratinocytes, monocytes and macrophages, and epithelial and endothelial cells [25,26], while the NO pathway in T lymphocytes, neutrophil, and mast cells is still debated [19,27]. Research in vivo shows that NO is mostly produced by T-helper 1 (Th1) lymphocytes, which inhibit Th1 response in an autocrine way, thus reducing interferon-γ (IFN-γ) production and simultaneously promoting Th2 response through IgE production enhancement [28]. Research on the effects of NO on the immune cascade does not reach this conclusion: some studies both in vivo and in humans demonstrate that NO inhibits Th1 and Th2 pathways equally [29,30]. Collectively, NO probably acts as on–off switch for inhibition or proliferation of T-cells, considering that at low concentrations it inhibits T-cell growth, while at high levels it might induce apoptosis [30]. The primary sources of NO in the airways remain epithelial cells and macrophages during Th2 inflammation [30]. Therefore, nNO assessment has become a matter of study in several pathologies of the nose and paranasal sinuses [13,16,18,31,32]. Apart from the enzymatic synthesis pathways, endogenous production of NO can occur through other non-enzymatic processes, which are not as thoroughly understood. For instance, NO can be generated in vivo by the reduction of nitrate to nitrite, a process that may be performed by bacteria [13,33].

### 2.2. Sampling and Measurements Methods

Measurement of FeNO has been a widely used and standardized method to detect eosinophil inflammation in lower airways diseases, particularly asthma [32,34]. In contrast, several instruments have been proposed to measure nNO, with different sampling techniques and analytical methods [32,34]. In this regard, chemiluminescence and electrochemical and laser sensors are currently available. The chemiluminescence method is the gold standard for nNO analysis as it is highly sensitive, with a very low detection threshold and fast response time, although it remains in use solely for laboratory analysis due to the high cost. Electrochemical sensors are the most used instruments for measuring nNO because they are more economic and portable compared to the other instruments [35,36,37].

**Figure 1:**
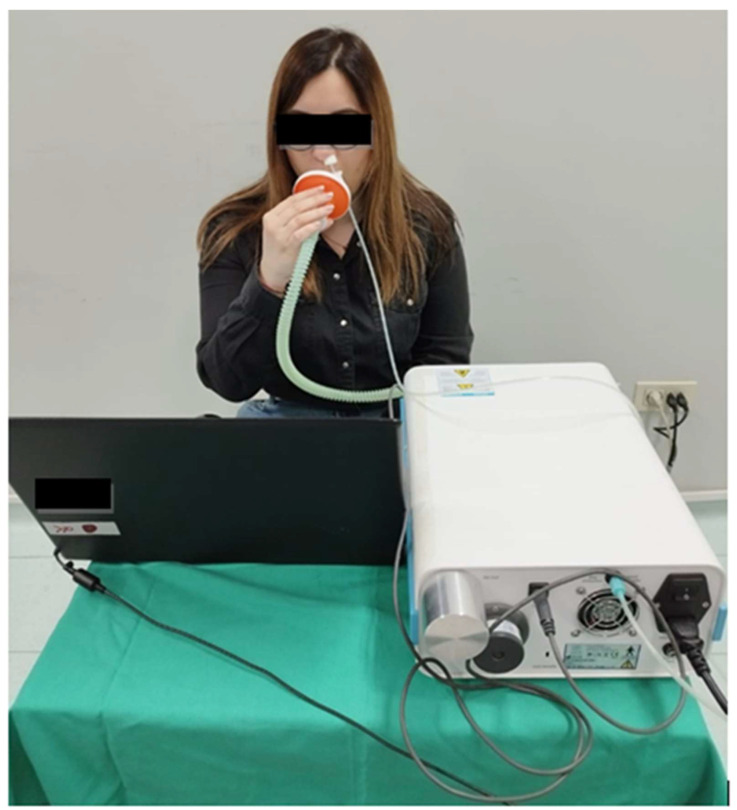
Measurement of nasal nitric oxide by electrochemical sensor in series sampling. NO is measured by using a nasal olive in one single nostril during tidal breathing.

All the instruments enable nNO sampling, whether in parallel or in series [32,34]. In the first case, NO is collected during exhalation from the chest using a mask covering both nostrils. In the second one, NO sampling is achieved by using a nasal olive in one single nostril [34] (Figure 1). The series sampling method is recommended by the American Thoracic Society and the European Respiratory Society using an aspiration flow rate between 250 and 3000 mL/min [32,34]. nNO measurement has to be stable to be compared among different subjects and, for this reason, the NO plateau should be achieved rapidly using high aspiration rates [32,34]. The aspiration should be at a constant flow rate from one single nostril with gas inflow in the other one and, using the velum closing maneuver, contamination from lower airways is abolished [34]. The nNO measurement fits for children older than 4 years old and adults who are able to cooperate to ensure velum closure [1]. When concomitant nasal polyps, sinusitis, or marked ostial obstruction occur in AR, nNO would not be detected as expected [1,38]. Given the above, it is expected that the nasal mucosa should not exhibit significant edema. In fact, obstruction of the osteo–meatal complex can lead to reduced levels of nNO, as it hinders the release of NO from the paranasal sinuses, where it is normally deposited [13,38]. 

Therefore, a new method to assess the patency of the osteo–meatal complex has been proposed by using nNO during humming, which is the production of a tone without opening the lips or forming words. Under normal conditions, humming causes a strong increase in nNO (humming responder), while, in the presence of obstruction of the osteo–meatal complex, this maneuver does not cause any increase in NO (humming non-responder) [39,40].

## 3. Nitric Oxide and Allergic Rhinitis: Clinical and Functional Mechanisms

nNO has been studied in different clinical diseases of the upper airways, being a potential tool in diagnosis and monitoring AR in both adults and children [41,42].

In AR, as with FeNO in asthma, nNO appears to be related to the degree of eosinophilic inflammation [43], as it comes from a Th2 inflammatory cascade and its production depends on allergen exposure [43]. After intranasal allergen exposure, nNO decreases in the first 20 min, later increasing after about 7 h and peaking after 24 h [44]. 

Using the same analyzer (Niox^®^ Mino, Aerocrine AB, Solna, Sweden), the same flow rate (0.3 L/min) and the same method (breath hold), two authors reported similar cut-off values (169.4 and 161.4 nL/min) with good specificity and sensitivity for nNO in AR [1,45,46]. Using other analyzers (Nano Coulomb^®^ Breath Analyzer, Sunvou-CA2122, Wuxi, China) instead, other authors reported cut-off values in AR and in healthy control subjects of 684.2 and 355.4 ppb, respectively [47].

According to the studies available in the literature, nNO levels have been found in individuals with RA to be higher than in non-RA controls. This was confirmed by a recent meta-analysis from our group, which consistently indicated that AR is associated with increased nNO levels when measured by both aspiration and expiration methods for perennial and seasonal disease [48]. In this meta-analysis, patients with seasonal AR exhibited increased levels of nNO as compared to controls only during the exposure to the allergens. This can be considered indirect evidence that the production of nNO in the nasal mucosa of RA patients is triggered by allergen exposure and subsequent inflammation, with an increased expression of iNOS in epithelial cells [49,50]. Furthermore, AR patients present an elevated nNOS immune reactivity around mucosal glands [51], as well as an overexpression of eNOS in the mucosal epithelium [52]. Therefore, a relationship between the increased expression of the different isoforms of NO synthase and the anatomical damage of the nasal mucosa in AR has been hypothesized [53].

Further investigation is still warranted to explore the relationships between various NOS isoforms and the extent of mucosal damage in AR [54]. To date, high levels of nNO in AR appear to be related to nasal mucosal damage, such as lack of vibrating cilia and basement membrane alterations, including absence of tight junctions with increased intercellular space [53]. Among all inflammatory molecules, NO modulates leukotriene B4 (LTB4)-induced neutrophil recruitment by changing rhinorrhea, thus indicating both a clinical manifestation of RA and a defensive mechanism [55]. nNO levels in AR patients seem to link even with symptoms severity because NO has effects on nasal mucosa [56], sneezing, and nasal leakage, even if some authors did not find this association statistically significant [57].

**Figure 2:**
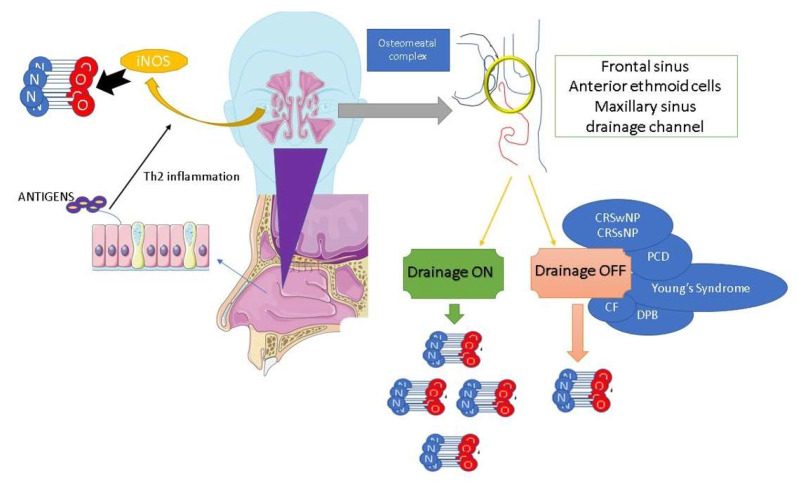
Nasal nitric oxide production and osteo-metal complex patency. NO: nitric oxide, iNOS: inducible nitric oxide synthase, CRSwNP: chronic rhinosinusitis with nasal polyps, CRSsNP: chronic rhinosinusitis without nasal polyps, PCD: primary ciliary dyskinesia, CF: cystic fibrosis, DPB: diffuse panbronchiolitis.

However, the increase in nNO in AR as compared to healthy controls is evident when there is no prominent obstruction of the paranasal sinus ostia, as the occlusion or blockage of the sinus ostia can impact the distribution of NO to the nasal cavity [58,59]. This variation in nNO distribution helps to explain the conflicting findings of certain studies that have suggested no significant difference in nNO levels between individuals with AR and healthy individuals [58,59]. Certain authors have examined nNO levels in relation to the opacification of the paranasal sinuses [43]. Their findings have shown a positive association between nNO and paranasal sinus opacification in patients with AR, particularly in cases without significant signs of chronic rhinosinusitis (CRS) according to the Lund-Mackay radiological staging system [43]. Therefore, the association between nNO and the inflammatory cascade in AR has become a matter of controversy in the literature. This is because the presence of nasal mucosa edema, which can hinder the patency of the paranasal sinuses, is a significant risk factor for CRS [43]. This is particularly relevant in cases of persistent AR, where nasal congestion persists for longer periods compared to intermittent AR [1,60]. Furthermore, when nasal obstruction at Visual Analogic Scale (VAS) score is lower than 7, or Nasal Airway Resistance (NAR) to airflow is lower than 0.65 Pa/cm^3^/s at anterior rhinomanometry, nNO could be considered as a real biomarker for AR and, for this reason, it may reflect nasal eosinophilic inflammation in patients only affected by AR with mild to moderate nasal obstruction [61]. On the other hand, in AR with severe nasal obstruction, identified by a VAS score higher than 7 or NAR higher than 0.65 Pa/cm^3^/s, nNO is not different from healthy controls [61]. In keeping with this, it is noteworthy that in cases where both the osteo–meatal complex and spheno-ethmoidal recess are obstructed, the inflammation and infection associated with CRS with (CRSwNP) or without nasal polyposis (CRSsNP) can lead to a decrease in the release of nNO from paranasal sinuses (Figure 2). This reduction in nNO release is significant as the paranasal sinuses serve as a reservoir of NO [43,56]. When comparing patients with AR and CRSwNP to patients with AR and CRSsNP, it has been observed that the former group tends to have lower nNO levels compared to the latter group, with a rapid increase in nNO observed after endoscopic sinus surgery [60,62]. Even if CRSwNP adult patients have high levels of iNOS in the nasal mucosa, it has been observed that nNO levels are decreased compared to those of non-complicated AR patients [38]. 

The evidence that measurements of nNO during humming is correlated with ostial function [39,40] has led to its potential use as test for osteo–meatal patency in AR, where humming does not cause any increase in nNO (humming non-responder). This method has been suggested as a suitable noninvasive test to assess the ostium patency and the effect of therapy in AR and in nasal polyposis [15,32,63].

## 4. Drug-Induced nNO Levels in Allergic Rhinitis

The topical application of L-NAME, a NOS inhibitor, has been found to decrease nNO production and prevent the increase in nasal airways resistance (NAR) induced by bradykinin, while partially inhibiting plasma extravasation mediated by platelet-activating factor (PAF), all mechanisms involved in AR [32,64].

Significant clinical evidence has emerged from the analysis of nNO levels after the administration of intranasal steroids (INS) and/or antihistamines (ATH) [65]. In particular, it has been observed that nNO levels may significantly decrease after topical treatment with these medications [65]. This decrease in nNO levels primarily reflects the effects of INS in reducing the expression of iNOS, thus highlighting the impact of INS on the regulation of NO production in the nasal mucosa [65]. In these patients, nNO was detected in the area of the inferior turbinate; in this part of nasal cavity, the metabolism of NO seems to be similar to that of bronchial mucosa in asthma [66]. It has been reported that nNO levels, blood eosinophils count, and severity of obstructive sleep apnea are higher in patients with persistent AR than in controls, and the administration of INS gives better results than ATH or leukotriene receptor antagonist (LRA) [67].

The levels of nNO in children with AR are influenced by their age, showing a positive association, which is likely explained by the increased development and pneumatization of the paranasal sinuses as children grow older [68]. In children with moderate-to-severe AR, higher nNO levels are associated with more severe nasal symptoms, as measured by VAS scores, and indicate greater severity of the disease, with a consequent decreased QoL for both patients and their caregivers [69]. In contrast, when these patients are treated with INS or ATH, a significant reduction in nNO levels and VAS scores for nasal symptoms should be expected, along with an improvement in QoL [69].

However, it is worth noting that several studies utilizing nasal sprays might encounter a potential limitation due to the presence of substances that could influence the levels of nNO [70].

## 5. Conclusions

The evaluation of nNO has shown potential as a useful biomarker in AR, as its levels increase in this condition and tend to decrease after treatment [71]. However, in order to study and monitor chronic inflammatory diseases of the upper airways in different settings (community, hospital, rehabilitation), it is crucial to establish a standardized method for sampling, analyzing, and reporting nNO measurements [72,73]. To date, the European Respiratory Task Force has recommended an electrochemical method for nNO in primary ciliary dyskinesia (PCD) screening [74], suggesting measurement while the patient exhales against resistance or performs trained velum closure to minimize dilution from the lower airways [72].

In the upper airways, NO acts as a biomarker of infections and inflammation, being produced by epithelial cells in the nose and so deposited in the paranasal sinuses [13,31,75]. Infectious diseases of the upper respiratory tract are associated with increased mRNA for iNOS in nasal cells, resulting in elevated levels of both FeNO and nNO as part of the immune defense response to viral infections [13,64,75].

According to the recent literature evidence, NO comes from eosinophilic inflammation in patients affected by AR, being detected only in the presence of mild-to-moderate nasal obstruction, with an uncomplete closure of paranasal sinuses ostia or NAR lower than 0.65 Pa/cm^3^/s [13,61]. Conversely, conditions that lead to reduced release of nNO from the paranasal sinuses into the nasal cavity, such as CRSsNP, CRSwNP, or anatomic alterations of the osteomeatal complex and/or spheno-ethmoidal recess, are associated with lower levels of nNO. For this reason, the above concomitant clinical conditions that induce reduced levels of nNO should always be taken into account when evaluating nNO in AR. Therefore, to appreciate the association between AR and high levels of nNO, patients should be selected after a clinical and instrumental evaluation aimed at studying nasal airflow and resistance by using anterior active rhinomanometry, paranasal ostia patency in nasal endoscopy, and CT scan, in order to identify potential clinical confounding factors.

In conclusion, additional research is still needed to assess the usefulness of this biomarker in monitoring Th2 inflammation and enhancing treatments across various clinical environments such as community, hospital, and rehabilitation settings [72,73]. Moreover, it is crucial to expand the literature to establish reliable cut-off values and a unique standardized procedure for nNO assessment.

## Data Availability

No datasets were generated or analyzed during the current study.
